# The complete mitochondrial genome of *Taxoblenus sinicus* Wei & Nie, 1999 (Hymenoptera: Tenthredinidae) and phylogenetic analysis

**DOI:** 10.1080/23802359.2021.1942271

**Published:** 2021-06-21

**Authors:** Zemin Sun, Meicai Wei, Gengyun Niu

**Affiliations:** College of Life Sciences, Jiangxi Normal University, Nanchang, China

**Keywords:** Mitochondrial genome, phylogenetic analysis, Allantinae, rearrangement

## Abstract

The complete mitochondrial genome of *Taxoblenus sinicus* Wei & Nie, 1999 was described. The circular genome is 15,878 bp with an A + T content of 80.4%. It contains 37 genes, and an 859 bp control region. The *trnI* (+)-*trnQ* (−)-*trnM* (+) cluster rearranges to *trnM* (+)-*trnQ* (−)-CR-*trnI* (+). Phylogenetic analysis demonstrates that Allantinae is a sister group of Tenthredininae and *T. sinicus* is one of the basal lineages of Allantinae.

*Taxoblenus* Wei & Nie, 1999 (Wei and Nie 1999) is a member of the subtribe Taxonina of the tribe Allantini of Allantinae. Species of *Taxoblenus* occur mostly in the alpine meadow region of Western China. The mitochondrial genome of *Taxoblenus* would be helpful to analyze the phylogeny of Allantinae. The complete mitochondrial genome of *Taxoblenus sinicus* Wei & Nie, 1999 was reported herein.

Samples of *T. sinicus* (CSCS-Hym-MC0193) were collected in Sigou, Zhugu, Menyuan, Qinghai, China (37.11 N 102.35 E) in July 2019. The specimen was deposited at the Asia Sawfly Museum, Nanchang (ASMN) (Meicai Wei, weimc@126.com) under the voucher number CSCS-Hym-MC0193. Whole genomic DNA was extracted from the thorax muscle of a female adult using the DNeasyR Blood &Tissue Kits (Qiagen, Valencia, CA). Genomic DNA was prepared in 150 bp paired-end libraries, tagged, and analyzed with the high-throughput Illumina Hiseq 4000 platform. A total of 4,734,909 raw reads (SRR14085847) were obtained. DNA sequences were assembled using MitoZ (Meng et al. 2019) and verified by Geneious Prime 2019.2.1 (https://www.geneious.com). Annotations of tRNAs were generated in MITOS web server (Bernt et al. 2013) and revised where necessary. The eleven unsaturated nucleotide sequences (*atp6*, *cob*, *cox1*, *cox2*, *cox3*, *nad1*, *nad2*, *nad3*, *nad4*, *nad4l*, and *nad5*) of 37 species were aligned using the MEGA7.0.26 (Kumar et al. 2016). The phylogenetic tree was constructed using RAxML (Stamatakis 2014) under the model GTR, and MrBayes (Ronquist et al. 2012).

The sequence yield by MitoZ was 15,325 bp and contained 37 genes with a partial control region (CR). The obtained sequences were thoroughly examined by reassembly using *Allantus togatus* (MW464859) and *Ferna bicoloricornis* (unpublished) as reference sequences (coverage were 6644 and 34,300, respectively). Reassembly using *trnI* and *trnQ* as references extended contigs and obtained a 126 bp overlap. After manual verification, we obtained a control region of 859 bp in length. To verify the reliability of the results, we used the control region itself as a reference, and received a high-quality mapping with the flanking tRNAs were found.

The circular genome is 15,878 bp, including 37 genes and a control region. The gene rearrangement only occurred in tRNA cluster as *trnM* (+)-*trnQ* (−)-CR-*trnI* (+). Except for *Asiemphytus rufocephalus* (incomplete), there are two existing gene rearrangement patterns in Allantinae. Due to the high diversity of Tenthredininae, the current sample coverage may not be sufficient to speculate the systematic significance of gene order patten. The A + T content of the whole mitochondrial genome is 80.40% (42.70% A, 11.80% C, 7.90% G, and 37.7% T), indicating significant A + T bias. Four start codons for protein-coding genes (PCGs) are used, ATG (*atp6*, *cob*, *cox3*, *nad2*, and *nad4*); ATA (*cox2*, *nad1*, *nad4l*, *nad5*, and *nad6*); ATT (*atp8* and *nad3*) and TTG (*cox3*). All PCGs use TAA as a stop codon except for *nad4* (T).

Phylogenetic tree based on mitochondrial genome sequences of 37 Tenthredinoidea species with two Xyelidae species as outgroup. The maximum-likelihood (ML) and Bayesian methods (BI) for phylogenetic analysis resulted in trees with similar topologies, and only the ML tree is shown in Figure 1. All conduction supports that (1) Allantinae is a sister group of Tenthredininae; (2) *T. sinicus* is one of the basal lineages of Allantinae (Wei and Nie 1998).

**Figure 1.**
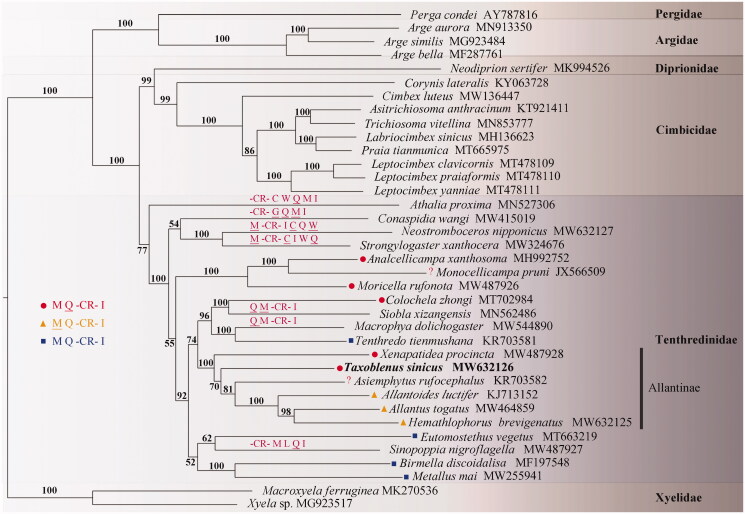
A maximum-likelihood (ML) tree based on the combined data of eleven unsaturated nucleotides. Numbers at the left of nodes are bootstrap support value. Partial gene orders are marked on the branches either directly or using the corresponding icons. The accession number for each species is indicated after the Latin name.

## Data Availability

The genome sequence data that support the findings of this study are openly available in GenBank of NCBI at https://www.ncbi.nlm.nih.gov under the accession number MW632126. The associated BioProject, SRA, and BioSample numbers are PRJNA714511, SRR14085847, and SAMN18318555, respectively. All related files had been uploaded to Science Data Bank (http://www.scidb.cn/s/pMvqE7v).

## References

[CIT0001] Bernt M, Donath A, Juhling F, Externbrink F, Florentz C, Fritzsch G, Putz J, Middendorf M, Stadler PF. 2013. MITOS: improved de novo metazoan mitochondrial genome annotation. Mol Phylogenet Evol. 69(2):313–319. 22982435 10.1016/j.ympev.2012.08.023

[CIT0003] Kumar S, Stecher G, Tamura K. 2016. MEGA7: Molecular Evolutionary Genetics Analysis Version 7.0 for bigger datasets. Mol Biol Evol. 33(7):1870–1874. 27004904 10.1093/molbev/msw054 PMC8210823

[CIT0004] Meng G, Li Y, Yang C, Liu S. 2019. MitoZ: a toolkit for animal mitochondrial genome assembly, annotation and visualization. Nucleic Acids Res. 47(11):e63. 30864657 10.1093/nar/gkz173 PMC6582343

[CIT0006] Ronquist F, Teslenko M, van der Mark P, Ayres DL, Darling A, Hohna S, Larget B, Liu L, Suchard MA, Huelsenbeck JP. 2012. MrBayes 3.2: efficient Bayesian phylogenetic inference and model choice across a large model space. Syst Biol. 61(3):539–542. 22357727 10.1093/sysbio/sys029 PMC3329765

[CIT0007] Stamatakis A. 2014. RAxML version 8: a tool for phylogenetic analysis and post-analysis of large phylogenies. Bioinformatics. 30(9):1312–1313. 24451623 10.1093/bioinformatics/btu033 PMC3998144

[CIT0009] Wei M, Nie H. 1998. Generic list of Tenthredinoidea in new systematic arrangement with synonyms and distribution data. J Cent South Univ. 18(3):23–31.

[CIT0010] Wei M, Nie H. 1999. A new genus and seven new species of Allantinae (Hymenoptera: Tenthredinidae) from China. J Cent South Univ. 19(3):9–14.

